# Cardiovascular magnetic resonance imaging in children after recovery from symptomatic COVID-19 or MIS-C: a prospective study

**DOI:** 10.1186/s12968-021-00786-5

**Published:** 2021-07-01

**Authors:** Gregory Webster, Ami B. Patel, Michael R. Carr, Cynthia K. Rigsby, Karen Rychlik, Anne H. Rowley, Joshua D. Robinson

**Affiliations:** 1 Division of Cardiology, Department of Pediatrics, Ann & Robert H. Lurie Children’s Hospital of Chicago, Northwestern University Feinberg School of Medicine, 225 E. Chicago Ave., Box 21, Chicago, IL 60611 USA; 2 Division of Infectious Diseases, Ann & Robert H. Lurie Children’s Hospital of Chicago, Chicago, IL USA; 3 Department of Radiology, Northwestern University Feinberg School of Medicine, Chicago, IL USA; 4 Department of Medical Imaging, Ann & Robert H. Lurie Children’s Hospital of Chicago, Chicago, IL USA; 5 Biostatistics Research Core, Stanley Manne Children’s Research Institute, Chicago, IL USA

**Keywords:** Pediatric, Cardiac magnetic resonance imaging, SARS-CoV-2, COVID-19, MIS-C

## Abstract

**Background:**

Cardiac evaluations, including cardiovascular magnetic resonance (CMR) imaging and biomarker results, are needed in children during mid-term recovery after infection with SARS-CoV-2. The incidence of CMR abnormalities 1–3 months after recovery is over 50% in older adults and has ranged between 1 and 15% in college athletes. Abnormal cardiac biomarkers are common in adults, even during recovery.

**Methods:**

We performed CMR imaging in a prospectively-recruited pediatric cohort recovered from COVID-19 and multisystem inflammatory syndrome in children (MIS-C). We obtained CMR data and serum biomarkers. We compared these results to age-matched control patients, imaged prior to the SARS-CoV-2 pandemic.

**Results:**

CMR was performed in 17 children (13.9 years, all ≤ 18 years) and 29 age-matched control patients without SARS-CoV-2 infection. Cases were recruited with symptomatic COVID-19 (11/17, 65%) or MIS-C (6/17, 35%) and studied an average of 2 months after diagnosis. All COVID-19 patients had been symptomatic with fever (73%), vomiting/diarrhea (64%), or breathing difficulty (55%) during infection. Left ventricular and right ventricular ejection fractions were indistinguishable between cases and controls (p = 0.66 and 0.70, respectively). Mean native global T1, global T2 values and segmental T2 maximum values were also not statistically different from control patients (p ≥ 0.06 for each). NT-proBNP and troponin levels were normal in all children.

**Conclusions:**

Children prospectively recruited following SARS-CoV-2 infection had normal CMR and cardiac biomarker evaluations during mid-term recovery. *Trial Registration* Not applicable.

## Background

Epidemiologic evidence gathered during the Coronavirus Disease 2019 (COVID-19) pandemic demonstrates that children infected with SARS-CoV-2 are relatively spared from severe disease compared to adults [1]. Most children infected with SARS-CoV-2 are asymptomatic or develop mild symptoms. However, some develop more severe symptoms including high fever, breathing difficulty and gastrointestinal disease. A small number eventually develop a rare but serious condition associated with COVID-19 involving multiple organ systems, referred to as Multisystem Inflammatory Syndrome in Children (MIS-C). As the pediatric effects of SARS-CoV-2 have become better understood, attention has turned to mid-term cardiovascular complications of acute infection and MIS-C.

Studies in adults have reported cardiovascular abnormalities using cardiovascular magnetic resonance (CMR) imaging. One to three months into recovery from COVID-19, CMR imaging was abnormal in 56–78% of middle-aged adults [2, 3]. Sequential evaluation of 26 college athletes demonstrated a 15% incidence of abnormal CMR imaging between 2 and 4 weeks after a positive serum test [4]. In contrast, a retrospective evaluation of 145 competitive athletes between 17 and 23 years of age demonstrated only a 1.4% incidence of abnormal CMR imaging during recovery [5]. These findings have been accompanied by abnormal serum biomarkers. Abnormal serum troponin was present in 4 young adults with negative CMR in the retrospective review [5] and cardiac serum biomarkers have remained elevated in a quarter of asymptomatic adults during recovery [2, 3].

Despite these data in adults, children recovering from acute symptomatic COVID-19 infection and MIS-C represent an under-studied group. We performed a prospective study of pediatric patients 1–3 months after symptomatic COVID-19 infection or MIS-C to assess cardiac involvement by CMR and serum biomarkers. Prospective CMR data were compared to age-matched control data obtained prior to the pandemic.

## Methods

### Participant identification and consent

Children with symptomatic COVID-19 or MIS-C were recruited between 10 and 18 years of age from September to December, 2020. We required at least one month to pass between the onset of acute infection and the study session. Children with pre-existing cardiac disease were excluded. Acute symptomatic COVID-19 cases were identified from prospective surveillance of children seeking care in our emergency department. Medical records were then reviewed to establish that potential participants had been symptomatic with a chief complaint consistent with COVID-19 and received a subsequent diagnosis of COVID-19 clinical disease based on a positive SARS-CoV-2 reverse transcription polymerase chain reaction result from a nasopharyngeal sample. We excluded children who tested positive for SARS-CoV-2 but had no symptoms or presented with minor symptoms (without fever, breathing difficulty or GI distress). An infectious disease specialist (ABP) and a pediatric cardiologist (MC) performed a systematic review of inpatient records for patients admitted with MIS-C. MIS-C cases were identified from children admitted to our institution who were diagnosed and treated for MIS-C based on the CDC case definition [6]. This included patients who presented with fever, systemic inflammation, multi-organ involvement with evidence of prior SARS-CoV-2 infection.

Family recruitment was initiated in 69 patients by a letter to the family’s home address, followed by phone follow-up (40 could not be reached or did not qualify after contact; 12 declined to participate). Consent was obtained, as approved by the Institutional Review Board. No contrast was administered because, in our institution, administration of intravenous CMR contrast excludes a minimal risk study status. Similarly, patients who could not perform CMR without sedation or anesthesia were excluded. A lower age limit of 10 years was chosen to ensure a high likelihood of successful completion of CMR without sedation. Symptoms were obtained by parent report using a semi-structured screening tool and validated by chart review. Recruitment sample size calculations were performed to detect a difference in 50 to 100 ms in T1 imaging with an alpha of 0.05 and a beta of 0.8. Participants were recruited in a prespecified 2:1 ratio of symptomatic COVID-19 to MIS-C to ensure that sufficient MIS-C patients were acquired within the study time and budget. At study completion, each participant received a $30 gift card.

### Cardiovascular magnetic resonance

#### Image acquisition

Studies were performed on a 1.5 T CMR scanner (Aera, Siemens Healthineers, Erlangen Germany). Conventional balanced steady state free precession (bSSFP) cine images were obtained in ventricular short- and long-axis planes. Myocardial native T1 maps were obtained using a free-breathing motion-corrected, electrocardiogram-triggered, modified Look-Locker inversion recovery (MOLLI) sequence with images acquired at end-diastole in the short axis plane at the left ventricular (LV) base, mid cavity, and apex [7]. Based on patient heart rate and body size, respectively, the number of recovery heartbeats were adjusted to allow for adequate T1 recovery between inversion pulses and the spatial resolution was optimized by reducing in-plane pixel size [8]. T2 mapping was performed using a free-breathing motion-corrected dark blood turbo spin echo sequence with a T2 preparation pulse and a bSSFP readout in the short-axis plane at the base, mid-chamber, and apex at end-diastole [9].

#### Image post-processing

Volumetric and functional data analysis was performed and myocardial T1/T2 values obtained using dedicated CMR post-processing software (Medis Suite MR, Medis Medical Imaging Systems BV, Leiden, The Netherlands). Endocardial and epicardial contours were traced on each myocardial map to exclude blood, epicardial fat, or artifact. The LV myocardium was divided into 16 segments as standardized by the American Heart Association [10] and T1 and T2 values for each segment were averaged to give a mean native global T1 and T2 value. The highest mean signal intensity from any myocardial segment was defined as the “segmental T2 maximum” value (T2 Max) [11]. All CMR images were interpreted by a cardiac radiologist and a pediatric cardiologist, and an additional independent review of all studies was performed, blinded to all clinical data, including disease state and outcome (JDR).

#### Serum biomarkers

Serum biomarkers were obtained on the same day as the CMR study. Serum N-terminal pro-B-type natriuretic peptide (NTproBNP) was performed on a Roche Cobas 8000 modular analyzer. The normal reference range (< 125 ng/L) was based on in-house studies on pediatric patients performed in conjunction with the Section of Cardiology, Department of Medicine, at the University of Chicago Medicine (as per Roche Diagnostics proBNP II STAT protocol). The lower limit of detection for troponin I levels at our institution was 0.3 µg/L, consistent with existing pediatric data [12]. Laboratory personnel performing the assay were blinded to the clinical status of the patients.

### Control data

In order to avoid using patients with subclinical SARS-CoV-2 infection in our control cohort, we identified a cohort of control patients by retrospective review of institutional CMR records. Referral diagnoses included chest pain with evaluation for coronary abnormalities, family history of cardiomyopathy, isolated electrocardiographic (ECG) abnormalities, and obesity with poor transthoracic imaging. To be included as control patients, CMR must have demonstrated normal cardiac structure, and normal biventricular size and global systolic function. If contrast was administered, there could be no evidence of late gadolinium enhancement (LGE). Patients could not have a history of any underlying systemic illnesses or drug exposures known to alter mapping values and patients must have been discharged from further cardiovascular follow-up with no evidence of cardiovascular disease. All controls were evaluated using the same pre-contrast protocol as recruited study patients, but all control patients had undergone CMR prior to 2019 to eliminate any possibility of SARS-CoV-2 infection.

### Statistical analysis

Mean and standard deviation (SD) were reported for normally distributed variables and median with interquartile range (IQR) for all others. A student t-test was used for univariate analysis of means. ANCOVA analysis (age co-variant) was used for analysis of means in global T1, global T2, and T2 Max. Data analysis was performed using STATA (Stata Corporation, College Station, Texas, USA).

## Results

### Demographics and symptoms

Children were recruited with symptomatic COVID-19 (n = 11, 65%) or MIS-C (n = 6, 35%). The mean age of participants was 13.9 years old (SD 2.2) and all participants were ≤ 18 years of age. Control patients were slightly older (16.8 vs. 13.9 years, p < 0.01). Sex was equally divided (8/17 (47%) girls). Self-reported race was 47% mixed or “other”, 35% White, 12% Black, and 6% Asian. 53% identified Hispanic ethnicity. The median time from acute symptomatic disease to CMR was 72 days (COVID-19) and 61 days (MIS-C). The IQR was 52–78 days for the combined population.

**Fig. 1:**
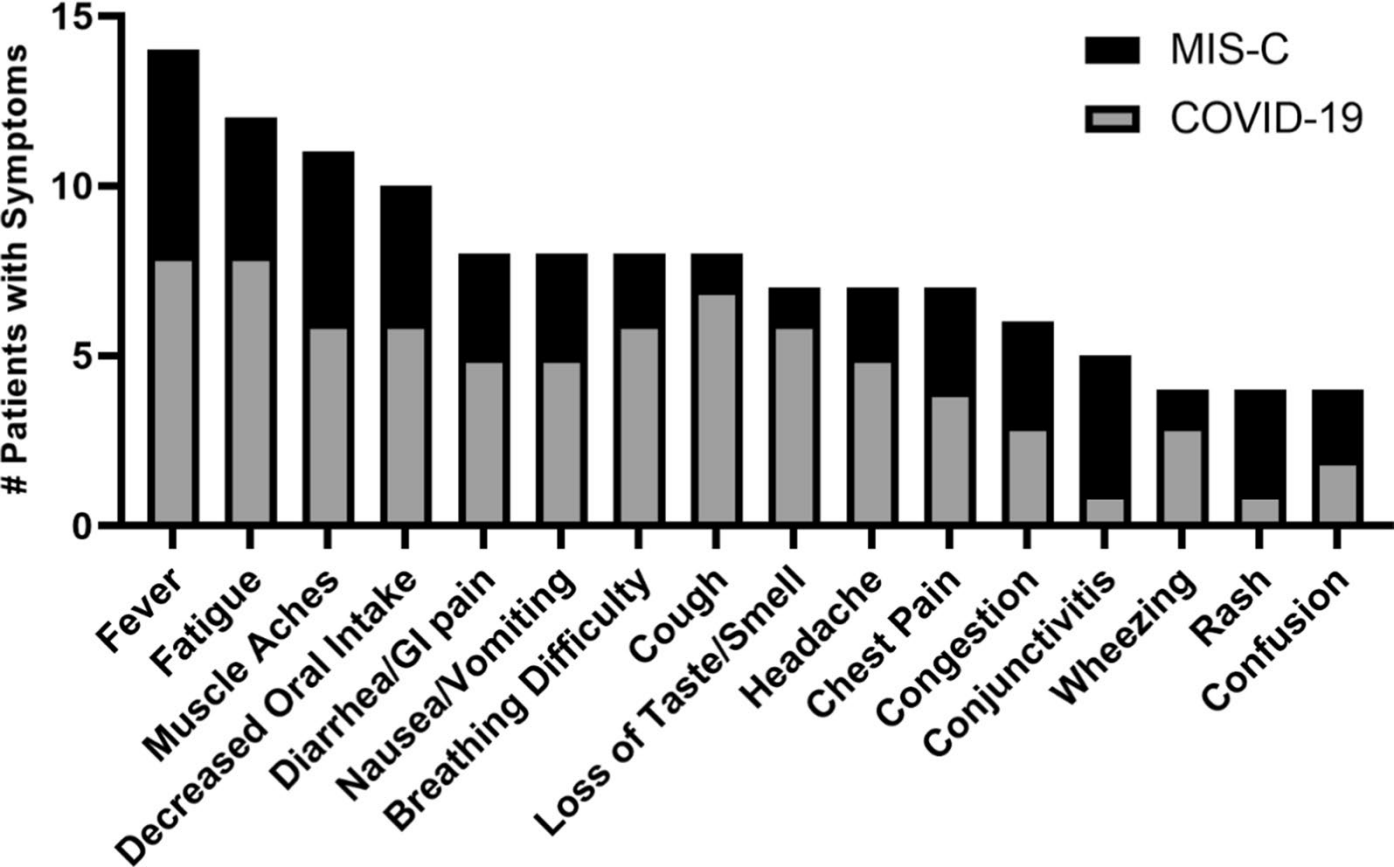
Parent report of prevalence of symptoms among cases. The ordinate axis displays the frequency of each symptom by parent report in participants with COVID-19 (n = 11) or MIS-C (n = 6). Parents were permitted to endorse all relevant symptoms and so the numbers do not equal the number of participants

Figure 1 tabulates the distributions of symptoms by family report. All symptomatic COVID-19 patients had fever (73%), vomiting/diarrhea (64%), or breathing difficulty (55%). All MIS-C patients were admitted to the hospital and received MIS-C-directed anti-inflammatory therapy. Mild to moderate LV dysfunction was present during hospitalization in 2/6 MIS-C participants. The minimum LV ejection fraction (LVEF) was 54% in one and 38% in the other. Mild coronary dilation was present in 2/6 (z-score 2.9 and 2.7). None had symptomatic heart failure. Function and coronary dimensions had normalized by echocardiography in all 4 patients prior to study CMR.

### Cardiovascular magnetic resonance

**Fig. 2:**
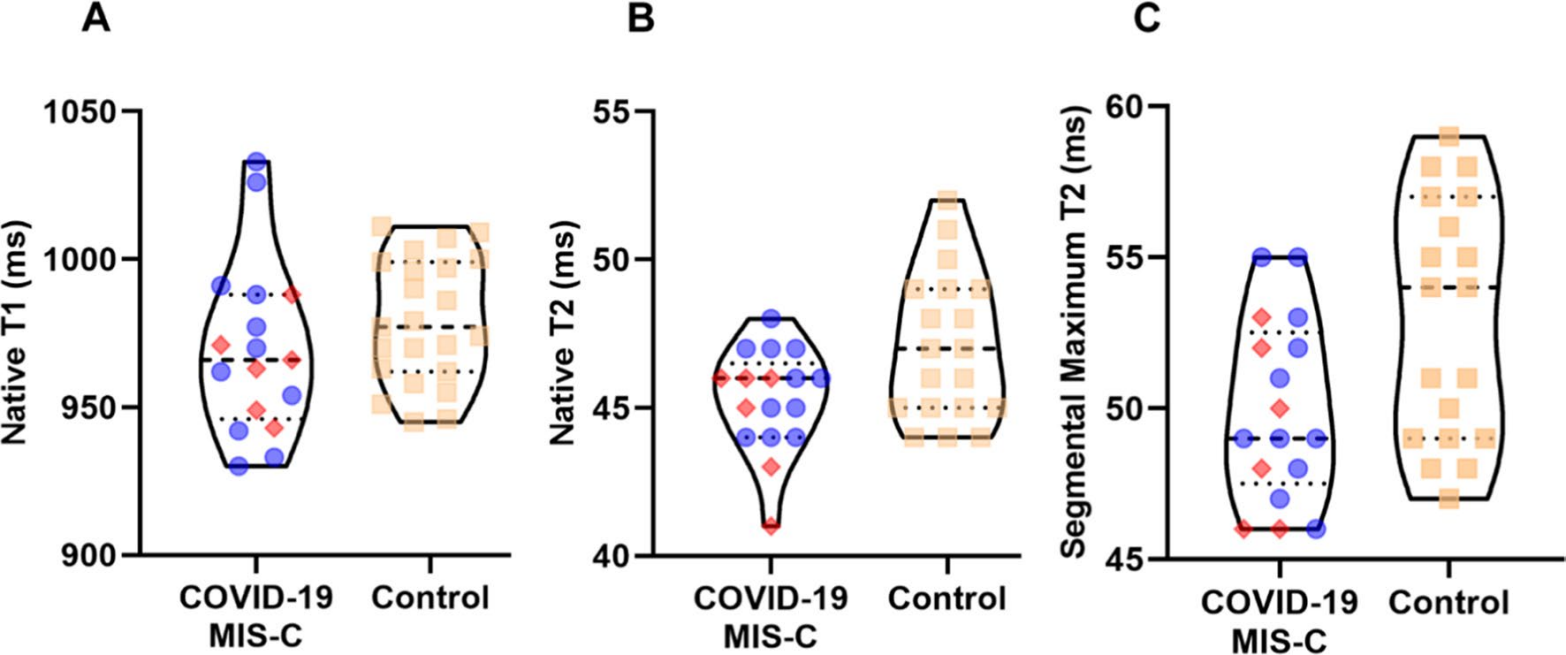
No CMR Abnormalities Were Present Following SARS-CoV-2 Infection in Children. Violin plots comparing CMR indices in cases and controls, with scatterplots overlaid. There was no difference between mean values in symptomatic COVID-19/MIS-C patients and control patients by **A** native global T1, **B** native global T2, or **C** segmental maximum T2 myocardial mapping. Blue circles = symptomatic COVID-19; red diamonds = MIS-C; tan squares = control patients; ms = milliseconds. Dashed horizontal line = median; dotted horizontal lines = quartiles

LVEF and right ventricular (RV) ejection fraction (RVEF) were indistinguishable between cases and controls (p = 0.66 and 0.70, respectively), although indexed RV and LV volumes were slightly higher in cases (Table 1). Mean native global T1 relaxation time, global T2 values and segmental T2 maximum values were not statistically different from control patients (p ≥ 0.06 for each, Fig. 2), nor were these values different between symptomatic COVID-19 and MIS-C patients (T1 global 973 vs. 963 ms, p = 0.52; T2 global 45.7 vs. 44.5 ms, p = 0.17; T2 segmental maximum 40.3 vs. 49.1 ms, p = 0.45). Biventricular size and function were normal in 16/17 participants. One 16 year-old boy with symptomatic COVID-19 had borderline biventricular dilation on CMR (LV end diastolic Z-score 2.1, RV 2.4), and normal biventricular function.

**Table 1:** Cardiac magnetic resonance imaging results for cases and controls

	Symptomatic COVID-19	MIS-C	All cases	Controls	p-value
Number	11	6	17	23	
Age, years	14.2 (2.4)	13.8 (2.2)	14.1 (2.2)	16.8 (1.3)	< 0.01
Height, cm	165.2 (18.1)	167.1 (10.9)	165.9 (15.6)	170.1 (10.4)	0.31
Weight, kg	69.6 (37.8)	76.8 (28.2)	72.1 (34.0)	73.3 (27.6)	0.90
BSA, m^2^	1.8 (0.5)	2.0 (0.4)	1.8 (0.5)	1.8 (0.3)	1.00
R EF, %	54.7 (5.8)	51.9 (3.9)	53.7 (5.2)	54.9 (11.3)	0.70
RV EDV/BSA**,** mL/m^2^	86.7 (17.4)	83.4 (14.1)	85.5 (16.0)	102.2 (15.2)	0.02
RV CO/BSA**,** L/min/m^2^	3.3 (0.7)	3.6 (0.5)	3.4 (0.7)	3.4 (0.7)	1.00
LVEF, %	57.4 (6.0)	55.9 (2.3)	56.9 (5.0)	56.2 (4.6)	0.66
LV EDV/BSA, mL/m^2^	84.7 (16.0)	80.2 (11.1)	83.1 (14.2)	98.5 (14.2)	0.02
LV CO/BSA, L/min/m^2^	3.4 (0.7)	3.8 (0.7)	3.5 (0.7)	3.5 (0.8)	0.97

*MIS-C* multisystem inflammatory syndrome in children, *RV* right ventricular, *LV* left ventricular, *BSA* body surface area, *EF* ejection fraction, *EDV* end diastolic volume, *CO* cardiac output, *min* minute. Standard deviation in parentheses. P-values were obtained by t-test comparing all cases against controls

### Serum biomarkers

The threshold for detection of troponin I in our laboratory was 0.3 µg/L. At the time of study CMR, all troponin I values were below 0.3 µg/L. Five of six patients with MIS-C had troponin I levels obtained during their hospitalization. The peak level was 0.56 and 0.39 µg/L in two patients and ≤ 0.3 µg/L in the other three patients. Only one COVID-19 patient had a troponin I level drawn at the time of symptoms and the level was 0.3 µg/L. Median NTproBNP was 22.8 ng/L (IQR 15.3–32.9) and all values were within the normal range (< 125 ng/L).

## Discussion

No significant cardiac disease was detected by CMR or serum biomarkers in this prospectively recruited pediatric cohort which included patients recovering from acute symptomatic COVID-19 infection and MIS-C. These findings contrast with 56%-78% of adults who exhibited cardiac abnormalities by CMR during recovery and up to a 15% prevalence in recovering college athletes [2, 3, 13]. CMR markers for edema (global and segmental maximum T2 values) and myocardial fibrosis (global native T1 values) were all normal.

Our data are important for three reasons. First, all participants in our COVID-19 cohort were symptomatic and presented to the emergency room with fever, vomiting/diarrhea, or breathing difficulty. All MIS-C patients had been hospitalized and 4 of 6 had cardiac abnormalities during hospitalization. Thus, participants from both study groups had an important history of clinical illness. Our data are most generalizable to children with significant clinical illness attributable to SARS-CoV-2 and are not diluted by asymptomatic children with positive viral screening studies.

Second, we provide a pediatric sample. Thus far, the contemporary literature from SARS-CoV-2 in young people has focused on college athletes and young adults [4, 5, 14]. Our study encompassed a truly pediatric cohort, between ages 10 and 18. Pediatric data are sparse and the only expert consensus statement about screening young people for potential cardiac involvement focuses on competitive athletes, who are only a subset of the young population [15]. Our data are most consistent with the low 1.4% incidence of myocarditis demonstrated on CMR during mid-term follow-up from Starekova and colleagues [5].

Finally, we performed a prospective study. We sequentially approached all patients from the emergency room and inpatient setting who met inclusion criteria. We believe that this approach minimizes the selection bias inherent in studying patients by retrospective data methods. Prospective recruitment remains the preferred methodology to accurately estimate the frequency of cardiovascular complications in children, without the selection bias introduced by retrospective data acquisition.

Starekova and colleagues recently reported on young adults recovering from COVID-19 with elevated troponin values. The authors wrote, “Future prospective studies that include serum laboratory tests, such as troponin levels […] are needed” [5]. We responded directly to that call by interrogating troponin I and NT-proBNP serum biomarkers in this pediatric population. None of the children in this study had elevated biomarkers during early recovery from either COVID-19 or MIS-C, a stark contrast with the 25% of adults who had abnormal troponin and BNP during the recovery phase of COVID-19 [2]. Moreover, we included a control group to avoid over-interpretation of CMR, as discussed in an editorial on this topic [16].

While we can only speculate how our results may have differed with a more comprehensive protocol including administration of gadolinium-based contrast agent, native T1 and T2 mapping have excellent diagnostic performance for myocardial injury in adult and pediatric myocarditis [8, 17], outperforming traditional Lake Louise criteria which include LGE. Similarly, in a prospective observational cohort of 100 adults who had been hospitalized and recently recovered from COVID-19, Native T1 and T2 had the best discriminatory ability to detect COVID-19-related myocardial pathology [3].

### Limitation

Our study is limited by small sample size. Additional studies are needed to exclude the possibility of a low frequency of long-term cardiac complications in children. In our study, the indexed RV and LV volumes in study patients were only slightly larger than in controls. There are two possible reasons for these mild discrepancies. First, controls were drawn from a population that was evaluated to exclude cardiac disease. This is a common methodology in pediatric CMR studies, but has a potential drawback: although none of the control patients received a diagnosis of cardiac disease, the incidence of subtle myocardial abnormalities may be higher in this group than in a randomly selected population cohort. Our control group probably represents the upper bound of normal LV and RV findings that are present in the population. To minimize this possibility, we verified that none of the case patients exceeded normative data previously established with a larger control group [8]. Second, our control population was slightly older than our study population. Though we controlled for body surface area and adjusted mapping sequence parameters for patient heart rate and size, subtle differences in myocardial mapping may occur with age. Despite these limitations, the subtle differences in cases and controls do not represent clinically important findings and no statistical differences existed in myocardial mapping values. Our results emphasize the importance of including control patients in CMR studies to avoid overinterpretation of findings in the cohort under investigation.

## Conclusion

Our study did not identify CMR abnormalities following SARS-CoV-2 infection in older children an average of 2 months into recovery from symptomatic disease.

## Data Availability

The datasets analysed during the current study are available from the corresponding author on reasonable request.

## Abbreviations

bSSFP: Balanced steady state free precession
CMR: Cardiovascular magnetic resonance
COVID-19: Coronavirus disease of 2019
ECG: Electrocardiogram
EDV: End-diastolic volume
IQR: Inter-quartile range
LGE: Late gadolinium enhancement
LV: Left ventricle/left ventricular
LVEF: Left ventricular ejection fraction
MOLLI: Modified Look-Locker inversion recovery
MIS-C: Multisystem inflammatory syndrome in children
NTproBNP: N-terminal pro-B-type natriuretic peptide
RV: Right ventricle/right ventricular
RVEF: Right ventricular ejection fraction
SARS-CoV-2: Severe acute respiratory syndrome coronavirus 2
SD: Standard deviation
